# CRISPR-Cas guide RNA indel analysis using CRISPResso2 with Nanopore sequencing data

**DOI:** 10.1186/s13104-024-06861-1

**Published:** 2024-07-26

**Authors:** Gus Rowan McFarlane, Jenin Victor Cortez Polanco, Daniel Bogema

**Affiliations:** 1 NSW Department of Primary Industries, Elizabeth Macarthur Agricultural Institute, Menangle, NSW 2568 Australia; 2 Sydney School of Veterinary Science, Faculty of Science, The University of Sydney, Camden, NSW Australia; 3 Catalina Stud, North Richmond, NSW Australia

**Keywords:** CRISPR, Cas, Cas9, gRNA, Nanopore, Sequencing, CRISPResso2, Indel

## Abstract

**Objective:**

Insertion and deletion (indel) analysis of CRISPR-Cas guide RNAs (gRNAs) is crucial in gene editing to assess gRNA efficiency and indel frequency. This study evaluates the utility of CRISPResso2 with Oxford Nanopore sequencing data (nCRISPResso2) for gRNA indel screening, compared to two common Sanger sequencing-based methods, TIDE and ICE. To achieve this, sheep and horse fibroblasts were transfected with Cas9 and a gRNA targeting the myostatin (*MSTN*) gene. DNA was subsequently extracted, and PCR products exceeding 600 bp were sequenced using both Sanger and Nanopore sequencing. Indel profiling was then conducted using TIDE, ICE, and nCRISPResso2.

**Results:**

Comparison revealed close correspondence in indel formation among methods. For the sheep *MSTN* gRNA, indel percentages were 52%, 58%, and 64% for TIDE, ICE, and nCRISPResso2, respectively. Horse *MSTN* gRNA showed 81%, 87%, and 86% edited amplicons for TIDE, ICE, and nCRISPResso2. The frequency of each type of indel was also comparable among the three methods, with nCRISPResso2 and ICE aligning the closest. nCRISPResso2 offers a viable alternative for CRISPR-Cas gRNA indel screening, especially with large amplicons unsuitable for Illumina sequencing. CRISPResso2’s compatibility with Nanopore data enables cost-effective and efficient indel profiling, yielding results comparable to common Sanger sequencing-based methods.

**Supplementary Information:**

The online version contains supplementary material available at 10.1186/s13104-024-06861-1.

## Introduction

The CRISPR-Cas systems have revolutionised biological research and biotechnology, offering a precise and user-friendly toolkit for genome editing [1]. At the heart of these systems are guide RNAs (gRNAs), which direct the Cas enzymes to specific sequences in the genome for editing [2, 3]. Understanding the efficiency of a gRNA at a specified target site and the types of editing outcomes it induces are crucial, particularly when creating targeted insertions and deletions (indels) through non-homologous end joining (NHEJ) events aimed at disrupting gene function [4–6].

Assessing the efficiency of gRNAs typically involves evaluating their capacity to introduce NHEJ indels at target sites. Profiling the frequency of each type of indel determines a gRNA’s ability to disrupt the function of a target gene [7]. While widely used, Sanger sequencing methods for gRNA indel analysis, such as Tracking of Indels by Decomposition (TIDE; [5]) and Inference of CRISPR Edits (ICE; [6]), are constrained by throughput and turnaround time. This has prompted a transition towards next-generation sequencing (NGS) approaches [8, 9].

Several NGS-based bioinformatic tools are available for analysing PCR amplicons spanning gRNA target sites for indel analysis in pooled populations, including CRISPR-GA [10], Cas-Analyzer [11] and the popular CRISPResso2 package [12]. However, these tools typically recommend Illumina or PacBio sequencing data as input, presenting constraints due to amplicon size limitations with Illumina platforms [13] or the high cost of PacBio sequencing [14]. Furthermore, Illumina and PacBio sequencing often necessitates external sequencing services and leads to delays in obtaining results.

Oxford Nanopore sequencing presents an alternative to Illumina and PacBio sequencing, offering theoretically unlimited amplicon size, cost-effectiveness and minimal capital requirements [15, 16]. Nanopore sequencing data has not typically been supported in NGS-based bioinformatic indel analysis tools for pooled populations due to lower sequencing quality [8, 10–12, 17]. However, recent advances by Oxford Nanopore technology to update its flow cell chemistry, pore engineering, and improvements to base calling software accuracy [18, 19], in conjunction with minor adjustments in CRISPResso2 command inputs facilitate the assessment of gRNA efficiency and indel frequency using Nanopore sequencing data in CRISPResso2.

In this research note, we highlight our method for screening gRNA efficiency and indel frequency. For simplicity, we refer to this method here as nCRISPResso2. However, the method does not require additional analyses outside of CRISPesso2. We validated this method by transfecting two Cas9 gRNAs into sheep and horse fibroblasts, targeting the myostatin (*MSTN*) gene [20, 21]. The *MSTN* PCR products amplified from these regions were > 600 bp using previously published primers [20, 21], making the amplicons unsuitable for Illumina sequencing. We compared our nCRISPResso2 results against TIDE and ICE to showcase the practicality and utility of nCRISPResso2 for gRNA indel analysis.

## Materials and methods

### Cell culture and transfections

Sheep and horse primary fibroblasts, obtained from previously deceased animals, were cultured in DMEM + 10% FBS. 1 × 10^6^ cells were transfected with 5 µg of pSpCas9(BB)-2 A-GFP (PX458; Addgene plasmid # 48,138; [22]) and 44 pmol of the respective single guide RNA (IDT; Supplementary Table 1 for crRNA sequences; [20, 21]) using a Neon Electroporator with settings of 1650 V, 10 ms and 3 pulses. Green fluorescent protein (GFP) positive cells were sorted using BD FACSMelody Cell Sorter (BD Biosciences) at 24 h post transfections to collect only transfected cells. Cells were cultured for an addition 24 h after sorting before harvesting for DNA extraction.

### DNA extraction and PCR

DNA was extracted from cells using a DNeasy Blood & Tissue Kit (Qiagen). PCRs were performed using 50 ng of gDNA with 2X Phusion High-Fidelity PCR Master Mix (NEB), 0.5 µM of each primer (IDT; Supplementary Table 1; [20, 21]), 3% DMSO and made up 50 µl with Nuclease free water. Thermocycling conditions can be found in Supplementary Table 2. PCR products were cleaned using QIAquick PCR Purification Kit (Qiagen) and used for both Sanger and Oxford Nanopore sequencing.

### Sanger sequencing and ICE and TIDE analyses

Cleaned PCR products were Sanger sequenced by the Australian Genome Research Facility. Chromatograms in ab1 format were used as input into the web browser interfaces of ICE (ice.synthego.com; [6]) and TIDE (tide.nki.nl; [5]) for analysis. Data was visualised with GraphPad Prism 9 (v9.3.0).

### Nanopore sequencing and CRISPResso2 analysis

Cleaned PCR products were prepared for sequencing using Oxford Nanopore Native Barcoding Kit 96 V14 kit (SQK-NBD114-96) before being loaded into an R10.4.1 MinION cell (FLO-MIN114) and run on a GridION device with MinKNOW (v23.11.7) and default settings, including Phred quality score (Q) ≥ 10 filtering. Reads were base called with Guppy superhigh accuracy mode (SUP, v4.2.0). CRISPResso2 (v2.2.14) was run on the resulting FASTQ files using commands described in Supplementary Table 3 using a high-performance computing system with 1.4 terabytes of random access memory (RAM) and an allocation of 32 processing cores. Indels with a frequency of less than 1% were excluded from analysis.

## Results

We conducted a comparative analysis of two widely used Sanger sequencing-based methods of gRNA indel analysis, ICE and TIDE, with nCRISPResso2. The Sanger sequencing chromatograms, utilised as input for ICE and TIDE, can be seen in the Supplementary Fig. 1 to 4. For the nCRISPResso2 analyses, 456,000 reads and 244,000 reads were obtained from Nanopore sequencing of sheep and horse *MSTN* amplicons, respectively. Within these reads, 78.5% of sheep basecalls and 60.5% of horse basecalls had a Q ≥ 20. Any nucleotides with Q < 20 were masked as ‘N’ using the built in CRISPResso2 function. After CRISPResso2 default alignment scoring (minimum 60% aligned bases) to the edge masked reference amplicon sequence, a total of 139,919 sheep reads (30.7% of total reads) and 47,360 horse reads (19.4% total reads) were used for indel analysis.

**Fig. 1:**
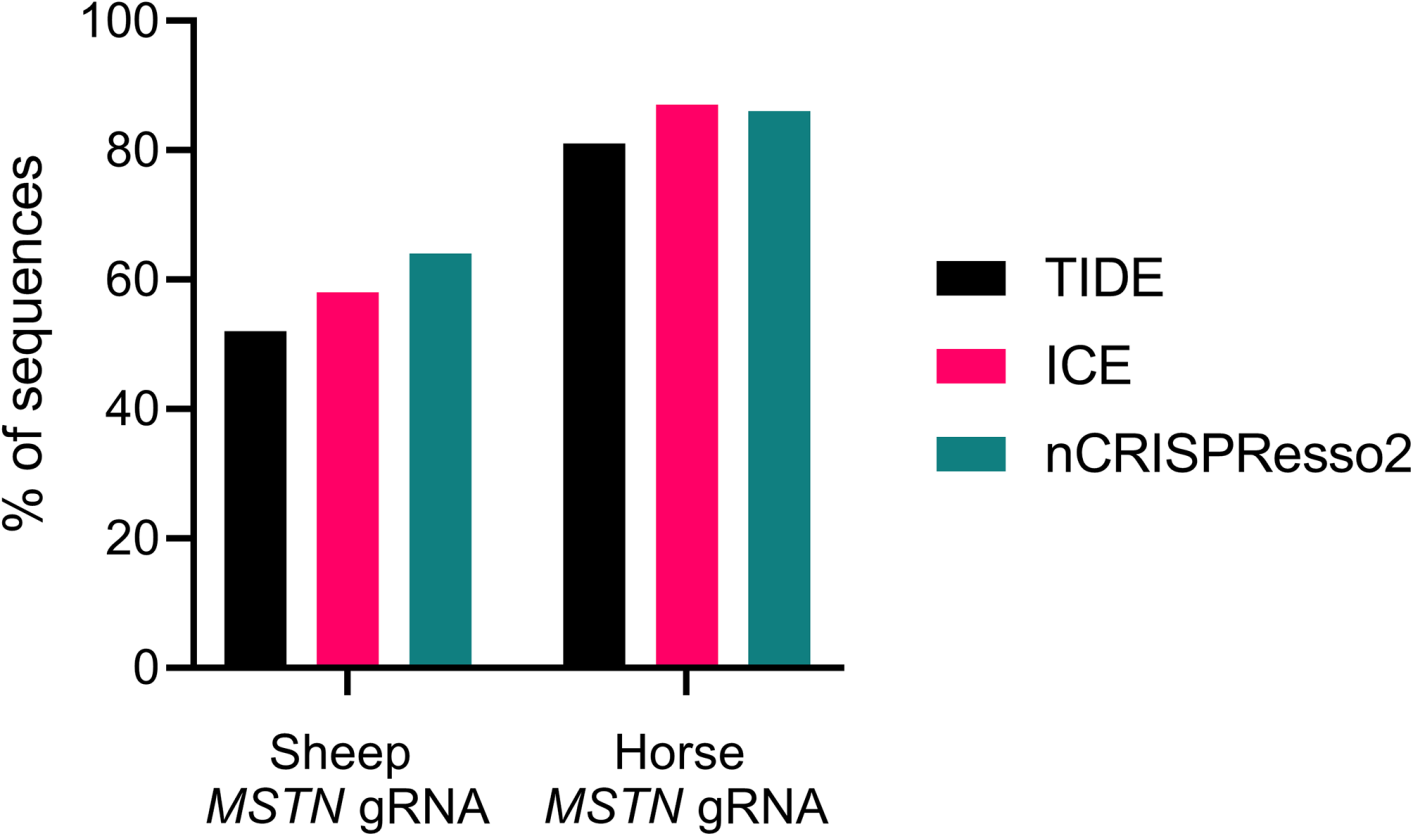
Percentage of amplicons with an indel. Fibroblasts transfected with sheep or horse *MSTN* gRNA were compared using TIDE, ICE and nCRISPResso2 methods of indel screening

Figure 1 shows that the overall indel frequency determined nCRISPResso2 corresponded to the results obtained from TIDE and ICE. For the Sheep *MSTN* gRNA, the percentage of amplicons with indels was 52%, 58%, and 64% for TIDE, ICE, and nCRISPResso2, respectively. For the Horse *MSTN* gRNA, 81%, 87%, and 86% of sequences exhibited indels when analysed by TIDE, ICE, and nCRISPResso2, respectively. Notably, ICE and nCRISPResso2 results were more concordant (7% variation) cross two experiments than between the ICE and TIDE Sanger sequencing-based methods (12% variation).

**Fig. 2:**
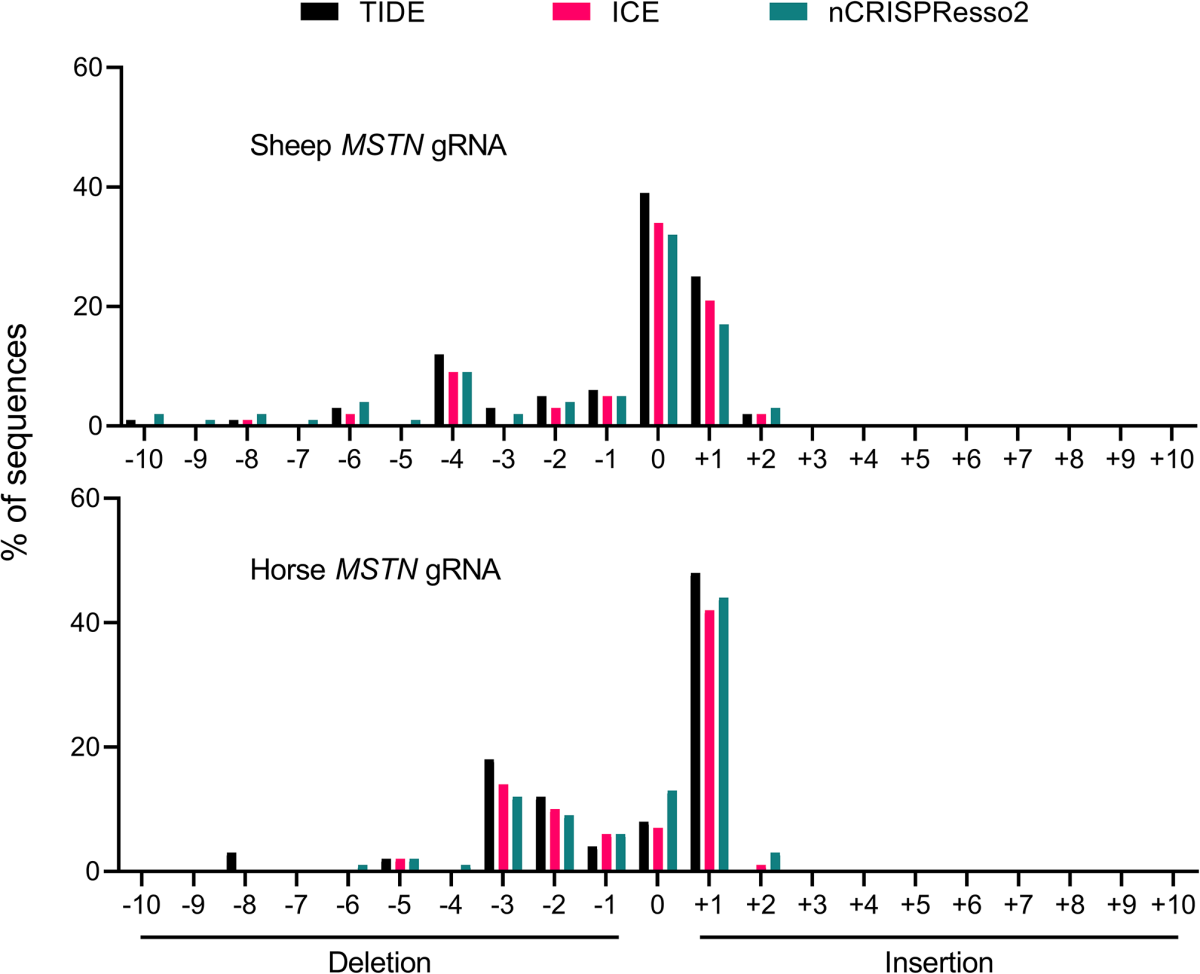
Indel frequency for the sheep and horse *MSTN* gRNAs. The frequency of each indel when analysed by TIDE, ICE and nCRISPResso2 for a 10 bp window upstream and downstream of the expected Cas9 cleavage site

Furthermore, as depicted in Fig. 2, the frequency of each demonstrated relative consistency among the three methods, with the top five most common outcomes being presented in the same order for TIDE, ICE, and nCRISPResso2 for both experiments. In sheep *MSTN* amplicons, the most common indels were + 1, -3, -2, 0, and − 1, while 0, + 1, -4, -1, and − 2 were the most frequent indels in horse *MSTN* amplicons. Similar to the overall percentage of indels, the comparison between nCRISPResso2 and ICE showed closer alignment in indel frequency than that between ICE and TIDE, with a 6% greater variation in collective indel frequencies observed across both experiments compared to the variation between CRISPResso2 and ICE results.

## Discussion

In this study, we examined the efficacy of CRISPR-Cas9 gRNA indel screening using nCRISPResso2 at the sheep and horse *MSTN* loci, employing PCR amplicons of 634 and 654 bp, respectively. Notably, the sizes of these amplicons exceed the compatibility threshold for Illumina sequencing, while PacBio sequencing remains prohibitively expensive [13, 14]. Nanopore sequencing offers a time- and cost-effective alternative for sequencing larger amplicons [15] that can be used as input in CRISPResso2 with minor command line adjustments.

One limitation of Nanopore sequencing is the noisy, lower-quality data it delivers compared to Illumina and PacBio platforms [17, 23]. To help mitigate this issue, we employed the latest Nanopore chemistry, flow cell design, and a super high-accuracy base-calling model to enhance data quality [18, 19]. Additionally, we set the built-in CRISPResso2 parameter ‘--min_bp_quality_or_N’ to 20, which masks base calls with a quality score less than 20, excluding poor-quality base calls with an accuracy of less than 99% from the indel analysis.

We observed differences in overall read counts and base calling quality when sequencing the sheep and horse *MSTN* amplicons. Variation in read counts is typical for barcoded Nanopore sequencing [24]. Although Oxford Nanopore’s latest software includes a beta version of barcode balancing to help address this issue [25], it was not used in this study. The reduced basecall quality in the horse sequencing is likely due to differences in the amplicon sequence, including a 50% increase in homopolymer stretches of 5 or more nucleotides compared to the sheep amplicon. These homopolymer stretches commonly pose a challenge for Nanopore sequencing technology [26].

CRISPResso2, which incorporates Needleman-Wunsch global alignment [27], typically encounters difficulties when processing noisy Nanopore sequencing data with variable read lengths and edge effects [12, 28]. To address these challenges and prevent edge effects from confounding our results, we masked the first and last 100 bp of the reference amplicon sequence. Nucleotides aligned to these masked regions are classified as substitutions and are excluded from the indel quantification.

Although masking the reference amplicons sequence in nCRISPResso2 helps address edge effects and variable read length, it limits the detection of larger indels. Masking 100 bp on each end of the reference amplicon classifies these base calls as unaligned and raises the homology requirement for the intervening sequence above the 60% nucleotide alignment default. For a read to be included in the nCRISPResso2 analysis, horse *MSTN* reads must have 86% homology for the intervening 434 bp sequence, or 88% homology for 454 bp of unmasked sheep reference sequence. This theoretically allows for the identification of indels up to 61 bp in horse amplicons and 53 bp in sheep *MSTN* amplicons. Therefore, reducing the minimum alignment score (--min_aln) below the 60% default or increasing the PCR amplicon length may be necessary for detecting larger indels using nCRISPResso2 with reference edge masking is adopted.

After implementing quality-based and reference edge masking, and setting CRISPResso2 to ignore substitutions (--ignore_substitutions), nCRISPResso2 provided indel results for horse and sheep *MSTN* gRNAs comparable to TIDE and ICE. Notably, nCRISPResso2 exhibited closer alignment with ICE results than with TIDE, or even between TIDE and ICE themselves. Given that the primary aim of this study is to identify Cas9 gRNAs capable of disrupting the horse or sheep *MSTN* gene through a frameshift, ignoring nucleotide substitutions was deemed appropriate. Additionally, it is important to acknowledge that the nCRISPResso2 commands used in this study are not suitable for identifying CRISPR-Cas-induced substitutions resulting from homology-directed repair or base editing enzymes.

Running nCRISPResso2 on the number of input reads used in this study requires access to a high performance computing system for efficient global alignment. Using 32 processing cores, we monitored RAM utilisation during the analyses, which did not exceed 5 gigabytes, and each analysis was completed within 1 h. Using fewer input reads, which can be cost-effectively achieved with a smaller Oxford Nanopore Flongle flow cell, would reduce compute requirements and costs but lower sensitivity for detecting less abundant indels.

Our study demonstrates CRISPResso2’s compatibility with Nanopore amplicon sequencing using only minor modification of input parameters. We implemented quality-based and reference edge masking to enhance its utility for indel identification. While our focus has been on two specific gRNAs, this sequencing run analysed an additional 14 gRNAs successfully using nCRISPResso2. Due to confidentiality constraints, we omit this data but mention it to illustrate that multiplexing significantly reduces costs, enhancing scalability and affordability. nCRISPResso2 yielded results highly comparable to the Sanger sequencing-based ICE tool within the quantified editing window. We hope this study encourages the adoption of nCRISPResso2 by fellow genome editors to streamline indel analyses and reduce costs.

### Limitations

- Our study presents data from only two gene editing experiments in sheep and horse fibroblasts.
- We have only evaluated nCRISPResso2 using Oxford Nanopore v10.4.1 MinION flowcells.
- We have not assessed the compatibility of our CRISPResso2 input parameters with larger or smaller amplicon sizes.
- We have not tested TIDE, ICE, or nCRISPResso2 against ratio dilutions of modified and unmodified DNA to determine the accuracy of each method in reflecting actual values.

### Electronic supplementary material

Below is the link to the electronic supplementary material.

Supplementary Material 1

## Data Availability

The datasets generated and/or analysed during the current study are available in the NCBI BioProject PRJNA1109565.

## Abbreviations

Cas: CRISPR-associated proteins
Cas9: CRISPR-associated protein 9
CRISPR: Clustered Regularly Interspaced Short Palindromic Repeats
crRNA: CRISPR RNA
GFP: Green Fluorescent Protein
gRNA: Guide RNA
ICE: Inference of CRISPR edits
Indel: Insertion or Deletion
*MSTN*: Myostatin gene
nCRISPResso2: CRISPResso2 using Nanopore data
NGS: Next-generation sequencing
NHEJ: Non-homologous end joining
PCR: Polymerase Chain Reaction
TIDE: Tracking of indels by decomposition
