# Supplementary material for: CRISPR-Cas guide RNA indel analysis using CRISPResso2 with Nanopore sequencing data
Source: BMC Res Notes. 2024 Jul 26;17:205. doi: 10.1186/s13104-024-06861-1 (PMC11282726; doi:10.1186/s13104-024-06861-1)
Supplement: Supplementary file 1 — Supplementary Material 1 [file 13104_2024_6861_MOESM1_ESM.pdf]

Supplementary material: CRISPR-Cas9 indel analysis using CRISPResso2 with Oxford Nanopore sequencing data

Authors: Gus Rowan McFarlane, Jenin Polanco and Daniel Bogema

Supplementary table 1. Nucleic acid sequences

Nucleic acid name	Sequence(PAM)	Application	Reference
Sheep MSTN gRNA	GGCTGTGTAATGCATGCTTG(TGG)	Input crRNA sequence for generating IDT sheep sgRNA	Crispo et al. (2015)
Horse MSTN gRNA	TGATGATTACCACGCGACGA(CGG)	Input crRNA sequence for generating IDT horse sgRNA	Moro et al. (2020)
Sheep MSTN F	TCACTGGTGTGGCAAGTTGT	Forward primer for sheep MSTN	Crispo et al. (2015)
Sheep MSTN R	TGCCCTCCTCCTTACGTACA	Reverse primer for sheep MSTN	Crispo et al. (2015)
Horse MSTN F	CCCAATTTTTGCCTTGGTGGT	Forward primer for horse MSTN	Moro et al. (2020)
Horse MSTN R	TTGTGCTGATTCTTGCTGGTC	Reverse primer for horse MSTN	Moro et al. (2020)

References

Crispo, M., Mulet, A., Tesson, L., Barrera, N., Cuadro, F., dos Santos-Neto, P., . . . Anegón, I. (2015). Efficient generation of myostatin knock-out sheep using CRISPR/Cas9 technology and microinjection into zygotes. PloS one, 10(8), e0136690.

Moro, L. N., Viale, D. L., Bastón, J. I., Arnold, V., Suvá, M., Wiedenmann, E., . . . Vichera, G. (2020). Generation of myostatin edited horse embryos using CRISPR/Cas9 technology and somatic cell nuclear transfer. Scientific reports, 10(1), 15587. doi:10.1038/s41598-020-72040-4

Supplementary table 2. PCR thermocycling conditions from both sheep and horse MSTN PCR.

Step	Temperature	Time	Cycles
Initial Denaturation	98 °C	30 seconds	1
Denaturation	98 °C	10 seconds	
Annealing	62.5 °C	20 seconds	32
Elongation	72 °C	25 seconds	
Final Extension	72 °C	5 minutes	1
Hold	10 °C		

Supplementary table 3. CRISPResso2 commands

Gene target	Command
Sheep MSTN	CRISPResso --fastq_r1 path_to_fastq_file -p 32 --plot_window_size 10 --min_frequency_alleles_around_cut_to_plot 1 --min_bp_quality_or_N 20 --ignore_substitutions -o path_to_output_directory -g GGCTGTGTAATGCATGCTTG --amplicon_seq NNNNNNNNNNNNNNNNNNNNNNNNNNNNNNNNNNNNNNNNNNNNNNNNNNNNNNNNNNNNNNNNNNNNNNNNNNNNNNNNNNNNNNNNNNNNNNNNNNNNAGGAAAAAGATTGTATTGATTTTAAAACCATGCAAAAACTGCAAATCTTTGTTTATATTTACCTATTTATGCTGCTTGTTGCTGGCCCAGTGGATCTGAATGAGAACAGCGAGCAGAAGGAAAATGTGGAAAAAAAGGGGCTGTGTAATGCATGCTTGTGGAGACAAAACAATAAATCCTCAAGACTAGAAGCCATAAAAATCCAAATCCTCAGTAAGCTTCGCCTGGAAACAGCTCCTAACATCAGCAAAGATGCTATAAGACAACTTTTGCCCAAGGCTCCTCCACTCCGGGAACTGATTGATCAGTACGATGTCCAGAGAGATGACAGCAGCGACGGCTCCTTGGAAGACGATGACTACCACGTTACGACGGAAACGGTCATTACCATGCCCACGGAGTGTGAGTAGTTCTGCTAGGGCAGAGCAACGACTNNNNNNNNNNNNNNNNNNNNNNNNNNNNNNNNNNNNNNNNNNNNNNNNNNNNNNNNNNNNNNNNNNNNNNNNNNNNNNNNNNNNNNNNNNNNNNNNNNNNN -n nhej
Horse MSTN	CRISPResso --fastq_r1 path_to_fastq_file -p 32 --plot_window_size 10 --min_frequency_alleles_around_cut_to_plot 1 --min_bp_quality_or_N 20 --ignore_substitutions -o path_to_output_directory -g TGATGATTACCACGCGACGA --amplicon_seq NNNNNNNNNNNNNNNNNNNNNNNNNNNNNNNNNNNNNNNNNNNNNNNNNNNNNNNNNNNNNNNNNNNNNNNNNNNNNNNNNNNNNNNNNNNNNNNNNNNNAAACACTAAATCTTCAAGAATAGAAGCCATAAAAATTCAAATCCTCAGTAAACTGCGCCTGGAAACAGCTCCTAACATCAGCAAAGATGCTATTAGACAACTTTTGCCCAAAGCTCCTCCACTCCGGGAACTGATTGATCAGTACGATGTCCAGAGAGATGACAGCAGTGATGGCTCTTTGGAAGATGATGATTACCACGCGACGACGGAAACAATCATTACCATGCCTACAGAGTGTAAGTAGTCCTGTTAGTGTATATCAACAATTCTGCTGACTGTTGTTCTAGTGTTTATGAGAAACAGATCTATTTTCAGGCTCTTTTAACAAGCTGTTGGCTTGTATGTAAGCAGGAAGGAAAAGAGTTTCTTTTTTTCAAGATTTCATGAGAAATTTACTAATGAGACTGAAATCTGCTGCATTATTTGTTTTCTTAGAGAGCTAAAAAGCTAAACANNNNNNNNNNNNNNNNNNNNNNNNNNNNNNNNNNNNNNNNNNNNNNNNNNNNNNNNNNNNNNNNNNNNNNNNNNNNNNNNNNNNNNNNNNNNNNNNNNN -n nhej

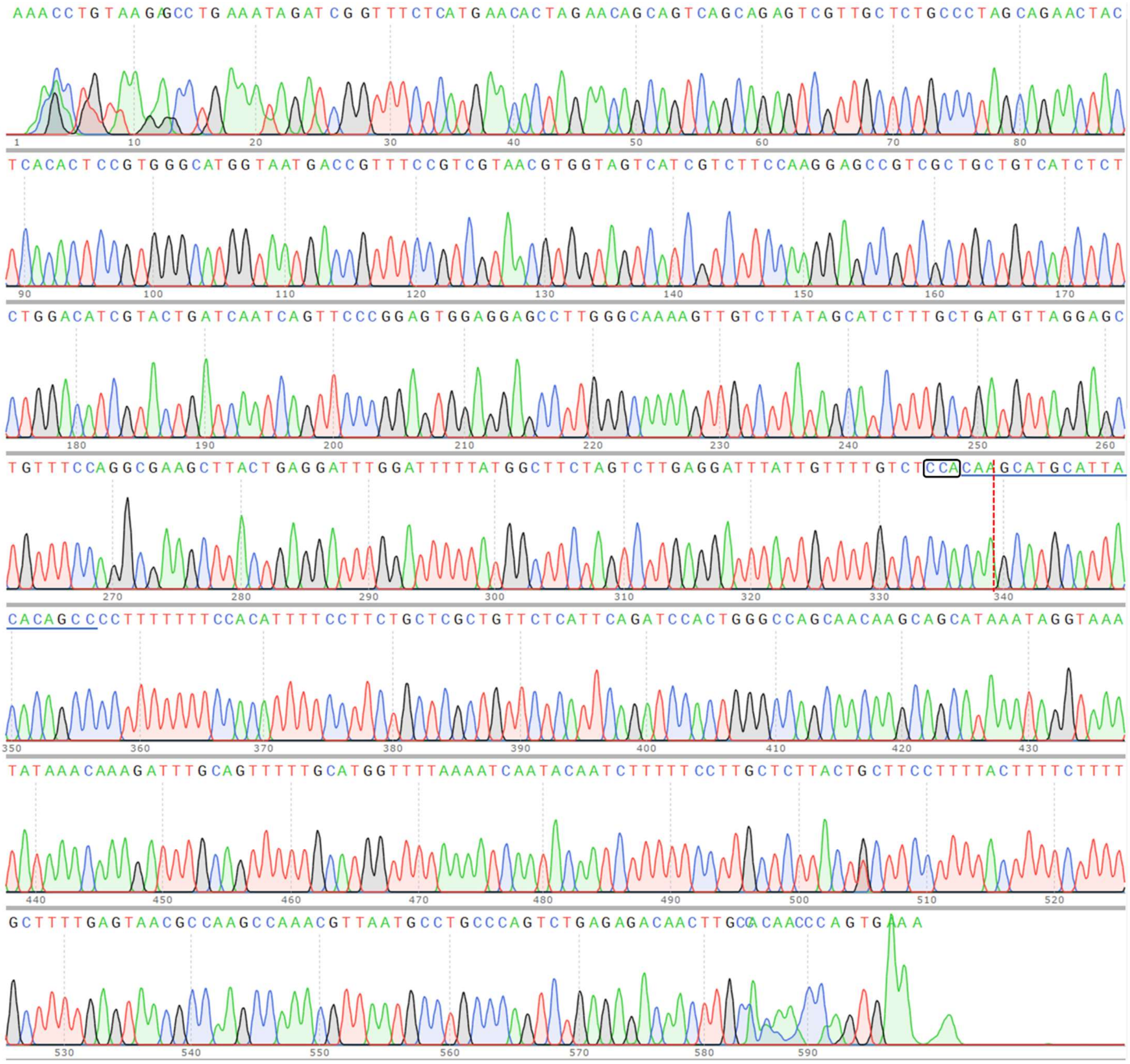

Supplementary figure 1. Sanger sequencing chromatogram depicting the MSTN PCR region in sheep control cells. Data was used as input for TIDE and ICE analyses. Blue line indicates crRNA. Black box highlights PAM sequence. Red dashed line shows Cas9 expected cleavage site.

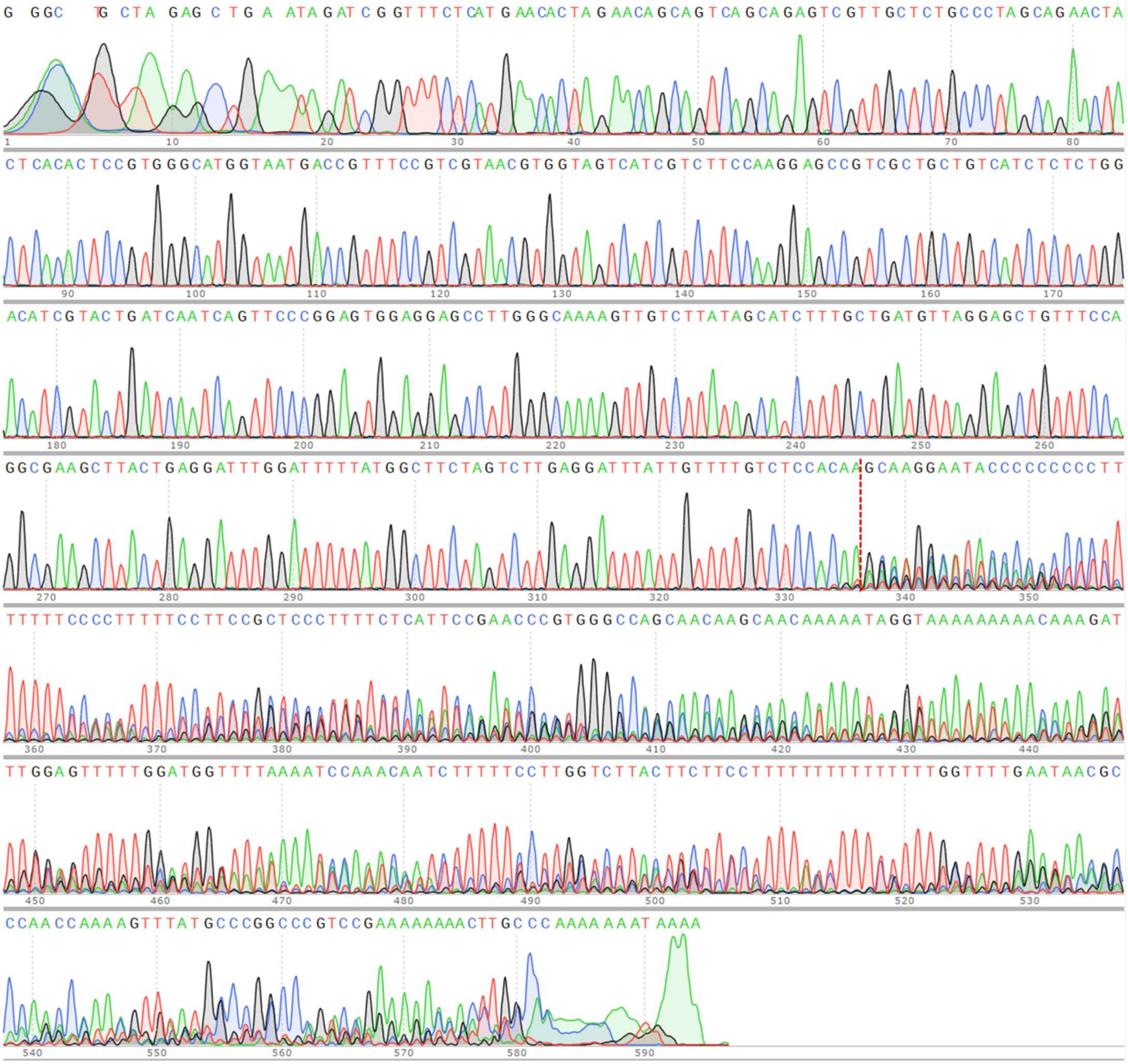

Supplementary figure 2. Sanger sequencing chromatogram depicting the MSTN PCR region in sheep cells transfected with Cas9/gRNA. Data was used as input for TIDE and ICE analyses. Red line indicates the expected Cas9/gRNA cleavage site.

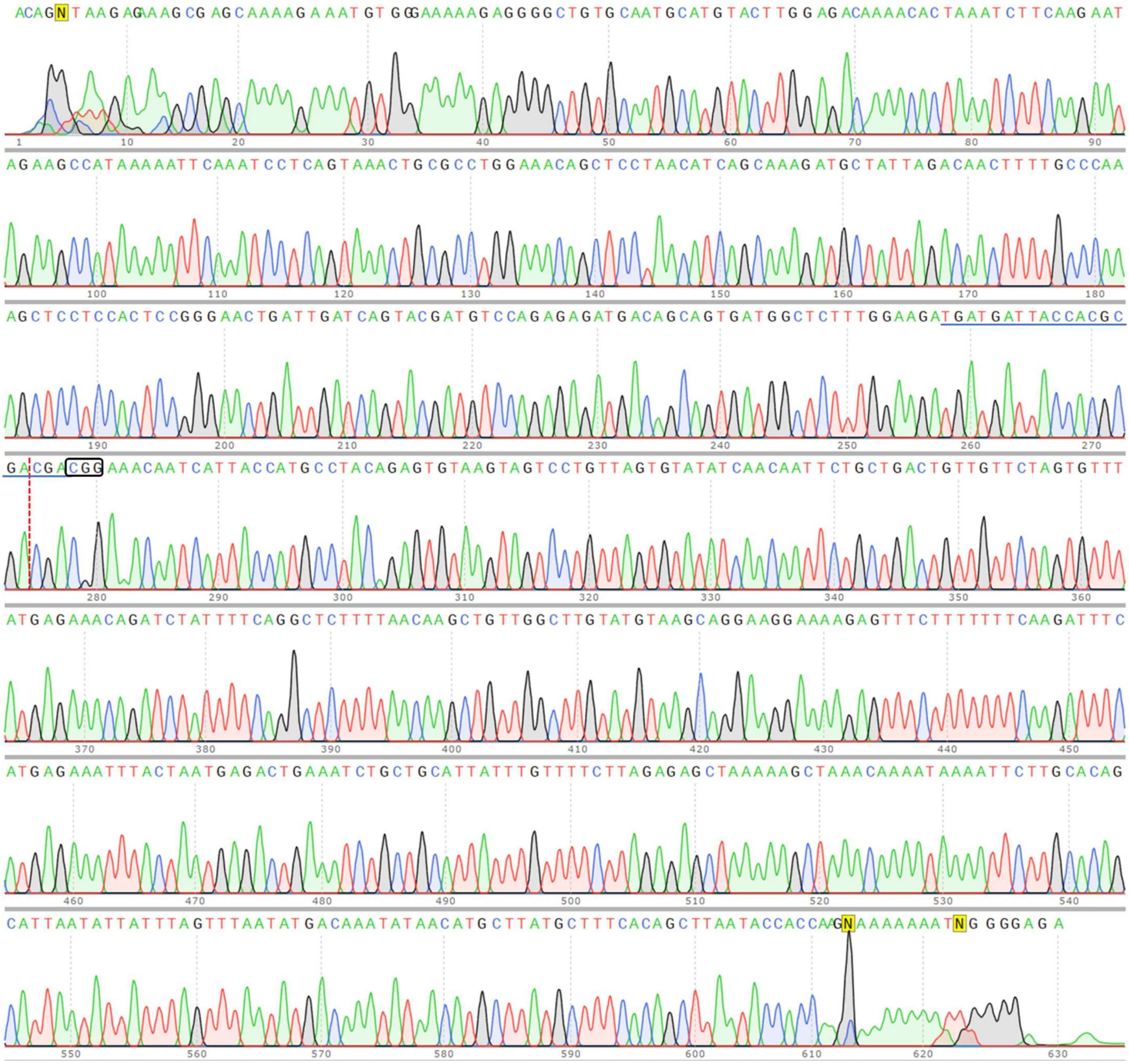

Supplementary figure 3. Sanger sequencing chromatogram depicting the MSTN PCR region in horse control cells. Data was used as input for TIDE and ICE analyses. Blue line indicates crRNA. Black box highlights PAM sequence. Red dashed line shows Cas9 expected cleavage site.

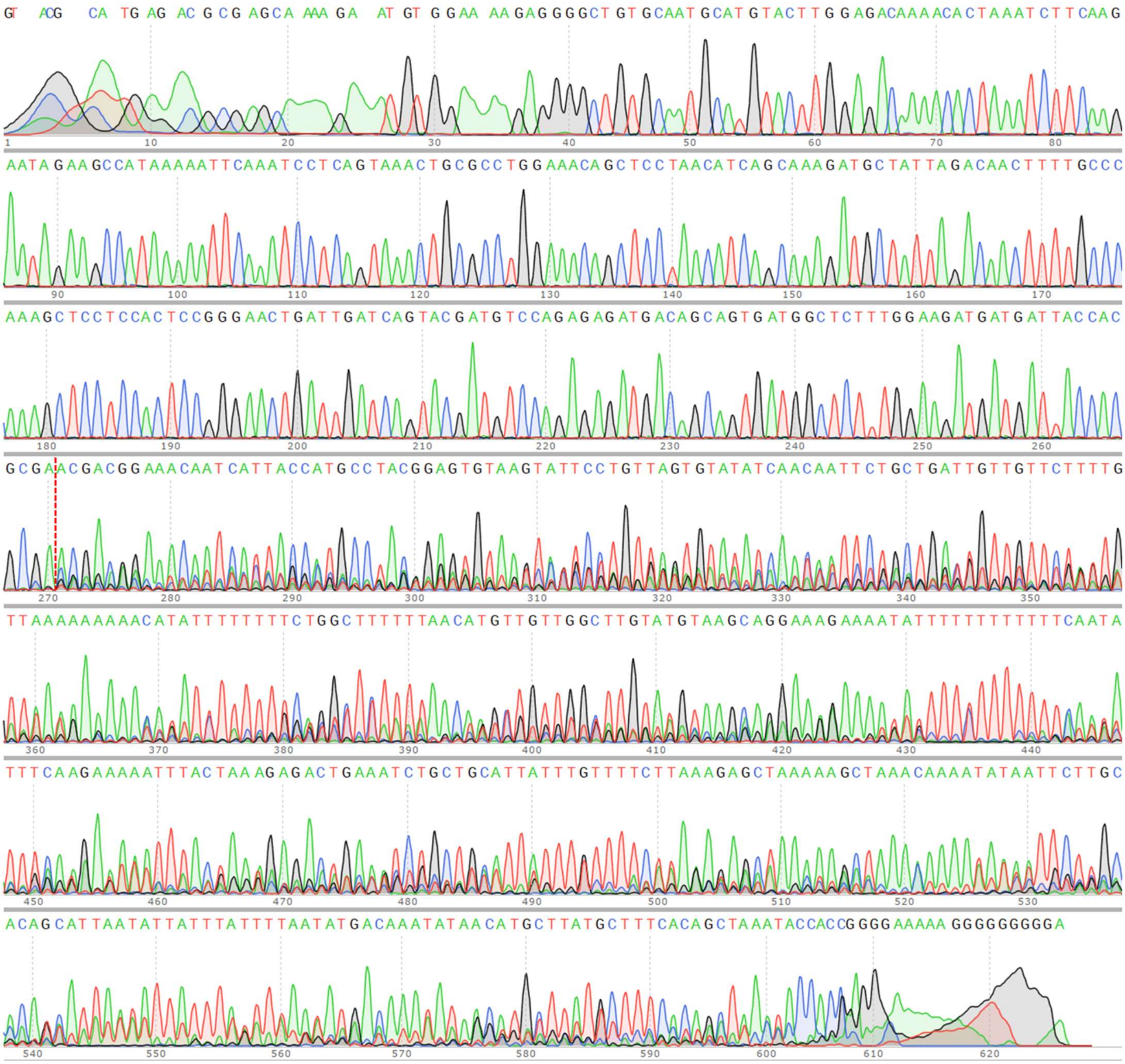

Supplementary figure 4. Sanger sequencing chromatogram depicting the MSTN PCR region in horse cells transfected with Cas9/gRNA. Data was used as input for TIDE and ICE analyses. Red dashed line shows Cas9 expected cleavage site.
